# Reflection points on how frailty concepts have changed clinical practice

**DOI:** 10.1016/j.tjfa.2025.100046

**Published:** 2025-04-17

**Authors:** Cornel Christian Sieber

**Affiliations:** a Director, Institute for Biomedicine of Aging (IBA), Friedrich-Alexander-Universität Erlangen-Nürnberg, Germany; b CMO, County Hospital Winterthur, Switzerland

## 1 Introduction

Frailty has long been recognized as an age-associated syndrome, reflecting a diminished resilience to internal and external stressors. Frail individuals face higher risks of hospitalization, functional decline, and mortality [1]. Over the past decade, however, our understanding of frailty has evolved, shifting from a deficit-based framework to a more resource-oriented approach that emphasizes intrinsic capacity (IC) and functional resilience.

As frailty has become increasingly embedded in clinical guidelines and decision-making, new challenges have emerged. The concept is now applied broadly, including in social, orthopedic, and oral frailty, leading to a lack of standardization. Simultaneously, frailty research has expanded, intersecting with sarcopenia, multimorbidity, and disability, necessitating a unified framework that better aligns with clinical realities and public health interventions [2].

## 2 Frailty and sarcopenia: "Sisters in content"

Frailty and sarcopenia are closely related conditions, often considered two sides of the same coin. According to the Fried Frailty Phenotype [3], frailty is defined by:

• Unintentional weight loss
• Self-reported exhaustion
• Weakness (low grip strength)
• Slow gait speed
• Low-level physical activity

Sarcopenia, in contrast, is primarily a neuromuscular condition involving loss of muscle mass, strength, and function [4]. The European consensus (2019) provides a well-defined structure for sarcopenia, yet frailty - in contrast to sarcopenia (M62.84) - still lacks an ICD code, complicating its use in clinical practice.

Recent research has demonstrated that nutrition and resistance training interventions can ameliorate frailty status and improve physical function, reinforcing the modifiable nature of frailty in older adults [5,6]. This highlights the importance of early screening and intervention to prevent functional decline.

## 3 Frailty in clinical and public health contexts

### 3.1 Frailty and clinical guidelines

Frailty is now integrated into international clinical guidelines [7,8], including the European Society of Cardiology (ESC) hypertension guidelines [9]. These guidelines state that while older adults should be treated similarly to younger individuals, frail patients should aim for a blood pressure range of 130–140 mmHg (systolic) and 80–90 mmHg (diastolic) if tolerated. In practice, this means higher tolerance for blood pressure-reducing therapies in frail persons - an appropriate "personalized" medical treatment approach.

### 3.2 Frailty and critical care: lessons from COVID-19

The COVID-19 pandemic underscored frailty as a key determinant of ICU outcomes [10]. The Clinical Frailty Scale (CFS) has been validated as a predictive tool for ICU admission and mortality [11]. Frailty was also used as a resource allocation criterion in triage settings, emphasizing its growing role in clinical decision-making.

### 3.3 The ethical dimension: "Facilism" and frailty discrimination

Unlike age and disability, frailty is not legally protected against discrimination [12]. This has led to the rise of "facilism", a form of bias against frail individuals, which often overlaps with ageism and ableism. As frailty research expands, it is crucial to address potential ethical concerns, ensuring that frailty assessments do not reinforce stereotypes or limit access to care.

### 3.4 From deficit-oriented to resource-oriented models

Historically, frailty has been synonymous with decline, a view that has influenced clinical practice and research. However, emerging frameworks emphasize IC, a WHO-endorsed concept that shifts focus from frailty as a deficit to frailty as a modifiable condition. IC encompasses:

• Cognition
• Vitality (energy/metabolism)
• Sensory function
• Locomotion
• Psychosocial resilience

A holistic, resource-oriented perspective enables the development of targeted interventions that enhance quality of life and delay dependency. When assessed through intrinsic capacity metrics, frailty becomes a dynamic, preventable condition rather than an inevitable outcome.

## 4 Conclusions and future directions

Over the past decade, frailty has gained widespread recognition, appearing in clinical trials, medical guidelines, and resource allocation frameworks. The challenge remains to standardize its definition, ensuring that frailty assessments integrate physical, psychological, and social dimensions. Emerging research underscores the importance of early interventions, particularly through physical activity, nutritional support, and personalized care plans. Future efforts should focus on integrating frailty into clinical practice as a resource-oriented concept, using frameworks like IC to guide assessment and intervention strategies.

Frailty is not just a medical condition—it is a multifaceted challenge that demands an interdisciplinary approach. As we refine our understanding, we must move beyond deficit-based models toward strategies that preserve function, independence, and dignity in aging populations.

## Declaration of competing interest

Cornel Sieber has no conflicts of interest to be disclosed.
